# Accuracy and Precision of Agents Orientation in an Indoor Positioning System Using Multiple Infrastructure Lighting Spotlights and a PSD Sensor †

**DOI:** 10.3390/s22082882

**Published:** 2022-04-09

**Authors:** Álvaro De-La-Llana-Calvo, José Luis Lázaro-Galilea, Aitor Alcázar-Fernández, Alfredo Gardel-Vicente, Ignacio Bravo-Muñoz, Andreea Iamnitchi

**Affiliations:** 1 Department of Applied Mathematics, Materials Science and Engineering and Electronic Technology, Rey Juan Carlos University, 28933 Móstoles, Madrid, Spain; 2 Department of Electronics, University of Alcalá, 28801 Alcalá de Henares, Madrid, Spain; josel.lazaro@uah.es (J.L.L.-G.); aitor.alcazar@uah.es (A.A.-F.); alfredo.gardel@uah.es (A.G.-V.); ignacio.bravo@uah.es (I.B.-M.); andreea.iamnitchi@edu.uah.es (A.I.)

**Keywords:** indoor positioning system (IPS), position-sensitive device (PSD), visible light positioning (VLP), angle of arrival (AoA)

## Abstract

In indoor localization there are applications in which the orientation of the agent to be located is as important as knowing the position. In this paper we present the results of the orientation estimation from a local positioning system based on position-sensitive device (PSD) sensors and the visible light emitted from the illumination of the room in which it is located. The orientation estimation will require that the PSD sensor receives signal from either 2 or 4 light sources simultaneously. As will be shown in the article, the error determining the rotation angle of the agent with the on-board sensor is less than 0.2 degrees for two emitters. On the other hand, by using 4 light sources the three Euler rotation angles are determined, with mean errors in the measurements smaller than 0.35° for the x- and y-axis and 0.16° for the z-axis. The accuracy of the measurement has been evaluated experimentally in a 2.5 m-high ceiling room over an area of 2.2 m${}^{2}$ using geodetic measurement tools to establish the reference ground truth values.

## 1. Introduction

Different techniques have been used for the development of indoor positioning systems (IPSs), many of which are complementary and can operate cooperatively, in order to achieve better positioning. Some of those techniques are based on computer vision [1]; radio waves, such as ultrawideband (UWB) [2] and radio frequency identification (RFID) [3]; ultrasound [4,5]; optical signals [6]; inertial measurement units (IMUs) [7,8,9] and radio frequency (RF) communications networks such as the global systems for mobile communications (GSM) and wireless local area networks (WLANs) [10]. The selection of which technology or particular system to use will depend on the application and/or the scenario where it is to be deployed. Factors such as area coverage, power consumption, cost, precision, accuracy and privacy will be relevant to determine which type of system to develop and which technologies to use [11,12,13].

There are different research works that make use of lighting based on light-emitting diodes (LED) as a means of information transmission (visible light communication—VLC); therefore, LEDs used in VLC systems have a dual functionality.

There are many works that can be found related to the use of light for communication, however, of particular interest here are those that use light from building illumination to develop indoor positioning and localization systems (VLP—visible light positioning-). In these, we include both dedicated to position and orientation determination. However, it is important to note that most of the work in IPS focuses only on position as the most relevant aspect, paying little or no attention to the orientation of the agents in the environment.

VLP systems have the advantage of using the infrastructure’s own lighting for positioning, so energy costs do not increase, and importantly, no new infrastructure or major infrastructure modification is needed to implement smart solutions. Moreover, VLP systems allow good accuracies while benefiting from the advantages of LED emitters such as electromagnetic immunity, high refresh rate and low cost.

The work addressed in this paper focuses on obtaining the orientation of the agent when the infrastructure signals are emitted by LED-based lighting systems and the sensor is placed in the mobile agent. To retrieve the orientation, it will be necessary that the PSD sensor (the type of sensor used in our research) on-board the mobile agent captures the signal coming from, at least, two different lighting lamps to eliminate any kind of indeterminacy.

The authors of this work belong to a research group that has a large trajectory developing IPS systems based on optical signals. Multiple localization systems based on the measurement of the phase of arrival (PoA) and angle of arrival (AoA) of the signal have been developed. Prior, the factors influencing sensor measurements were studied and proposals for electrical and geometrical calibration of our systems were developed. A comprehensive analysis and correction of distortions and procedure for modeling and correcting errors due to the multipath effect are also proposed in previous research.

In the following, we indicate the state-of-the-art of relevant works in the literature related to developments in both position determination (most numerous) and orientation determination.

Work [14] proposes using technology to emit their information of positioning to connected devices within the coverage area: lighting is used as a beacon and lighting.

In [15], an image sensor receives the positioning signal emitted from each LED bulb and provides localization measurement with an accuracy of about 1.5 m. In [16], the authors use an image sensor with a frame processing rate up to 48 kHz. The positioning information is decoded by the receiver knowing the impact point on the image sensor. In tests conducted in an area of 5.4 × 7.5 m${}^{2}$ with a height of 3 m the maximum horizontal plane error the system makes was as low as 10 cm.

Work in [17] performs positioning using four LEDs simultaneously. They achieve positioning errors of less than 10 cm.

The authors of [18] present an IPS based on received signal strength (RSS) from LEDs, improving the results of other works by using multiple photodiodes in the receivers. Positioning errors of 20 cm at a distance range of about 1.8 m is achieved. These systems have the disadvantage of RSS attenuation at greater distances and angles. The work in [19] proposes a system with infrared emitters (IRED) and an image sensor optimized for IRED beacon detection. In [20], an intelligent space was proposed to determine, by triangulation, the position of systems moving through the coverage area. Mobile agents emit signals in the non-visible range, allowing multiple photodiodes (detectors) located in the environment to obtain the angle of arrival and, by triangulation, know the position of the agent. For a test area of 7 × 2 m${}^{2}$, maximum errors of 70 cm are obtained; in this case, the system requires prior installation and calibration. The authors of [21] present a work based on three photodetectors and two IRED emitters to localize and position a robot in an indoor environment, obtaining an accuracy of 10 cm and 0.1 rad in the orientation angle for a mobile robot that moves at 0.2 m/s. In [22], using a system based on a QADA detector and four infrared LEDs, over a 2×2×2 m${}^{3}$ space, a positioning error of 10 cm and an estimation of the rotation angle with an average error of only 0.9° were obtained. We have not found any papers, other than those indicated above, that show the error made in the determination of the position of the agents.

Work [23] proposes an indoor 3D VLP and orienteering (VLPO) scheme. By using only two LEDs and two photo-detectors, they could determine simultaneous 3D localization and receiver orientation estimation efficiently. Further, to eliminate the location uncertainty caused by receiver tilt, they propose a location selection strategy which can determine the true location of the receiver. Results achieved a mean 3D positioning error of 7.4 cm and a mean azimuthal error of 7.0 deg. Moreover, this was achieved with 90.3% of 3D positioning errors less than 20 cm and 92.6% of azimuthal errors less than 5 deg.

In [24], the receiver of the IPS is based on a quadrant photodiode angular diversity aperture (QADA) plus an aperture placed over it. Encoding techniques are used to permit the simultaneous emission of four co-planar emitters and their processing in the receiver (3.4 m from the emitters). It uses Monte Carlo simulations to characterize the absolute errors for a grid of test points under noisy measurements, as well as the robustness of the system when varying the 3D location of one transmitter. The estimation of the receiver’s position for roll angles of the receiver of 0°, 120°, 210° and 300° achieves average absolute errors and standard deviations of 4.33 cm, 3.51 cm and 28.90 cm; and 1.84 cm, 1.17 cm and 19.80 cm in the coordinates x, y and z, respectively.

In [25], the authors indicate that by using a photodiode as a receiver in a VLP system, this will have a small rotation angle during movement, which will result in a massive positioning error ignoring the angle. In their work, the use of an improved whale optimization algorithm (IWOA) is proposed to reduce the error caused by the PD rotation. Authors indicate, the IWOA algorithm is efficiently utilized to address the problem. Simulation results show that when the PD has a rotation angle, the average positioning error estimated by ignoring the rotation angle is 27.14 cm, while that estimated by considering the rotation angle is 7.85 cm.

In [26], an AoA-based IPS was proposed considering that the target to be positioned moves in a plane. The target emits the signal and the position-sensitive device (PSD), located at a known position on the ceiling of the environment, obtains the position of the target.

The strengths of this system can be summarized as high accuracy, high precision and large coverage area. The disadvantages are that, in addition to the invasive installation of the PSD on the ceiling of the infrastructure, there are errors caused by the multipath effect and distortions that have an impact on the ideal AoA value. A procedure for modeling and correcting errors due to the multipath effect is also proposed in [27]. From these research works, it could be concluded that for a PSD-based IPS, the use of AoA obtains better positioning values than the use of PDoA (phase difference of arrival).

The work [28] analyzes the most suitable multiple-access discrimination techniques for the development of a visible light positioning (VLP) system based on PSD. It shows how the FDMA technique is the most suitable technique for PSD-based IPS, since it is able to discriminate between the emitters in the system with little or no interference.

In [6], a precision and accuracy evaluation of AoA-based IPS using a PSD and infrastructure lighting is presented. The IPS is assessed using different positioning approaches according to the number of visible transmitters (1, 2 or more LED emitters). A mean positioning error (accuracy) of 9.4 mm is obtained when simultaneous observations from two transmitters are used and the receiver moves in a plane. In case 4 emitter signals reach the receiver, the mean positioning error is below 49 mm, yielding a full 3D pose of the target without any additional measurements or prior assumption. In all cases, a precision (2σ) better than 6 mm for a measurement time of 10 ms is obtained.

## 2. Proposed Measurement System Based on PSD Sensors

**Figure 1:**
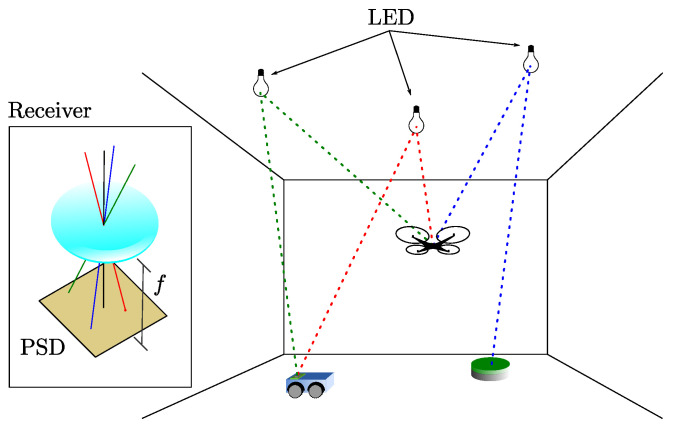
Scheme of the proposed positioning system.

The scheme of the proposed positioning systems is shown in Figure 1. The light sources based on LED emitters are placed at known fixed positions on the ceiling, and each emitter sends a modulated signal that allows its identification using a frequency division multiple access (FDMA) scheme. PSD sensor receives the signals simultaneously, taking into account the optical system attached to the PSD, the AoA of each emitter signal is calculated. It should be noted that for this work, the emitters should be considered point-like and with a Lambertian radiance pattern with rotational symmetry, which makes their orientation not relevant for the measurements of the AoA. Our only requirement is the lamps emit a different pattern from a collimated beam, since in that case it would only be captured from one position.

Figure 1 shows schematically some of the possible applications. A terrestrial robot that moves on a plane and needs only the signal from two emitters, a robot that has integrated sensors (e.g., IMU or odometry) to know its rotation and therefore would only need the signal from one emitter, and finally an unmanned aerial vehicle (UAV) that needs to know its position and angles in 3D and would need the signal from three or more emitters.

**Figure 2:**
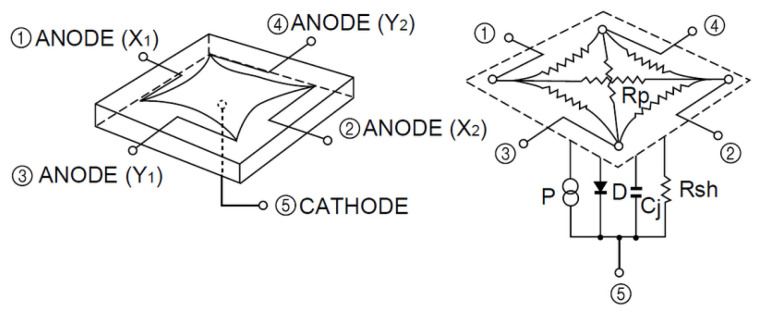
Equivalent circuit of the PSD pin-cushion (image courtesy of Hamamatsu, obtained from the PSD technical information).

The receiver used in this work is a PSD sensor consisting of four anodes and a common cathode. We have attached a lens to confine the light from each of the emitters to a point of light on the surface of the PSD sensor as shown in Figure 1. Figure 2 shows the equivalent circuit of a two-dimensional pin-cushion PSD sensor.

PSD sensor allows us to calculate the impact point of the signal on the PSD surface as a function of either the four electrical currents or voltages obtained from the currents at each PSD anode. The Equations (1) and (2) show such relation.

(1) $$\begin{array}{c} x=\frac{L_{X}}{2}\frac{\left(V_{X2}+V_{Y1}\right)-\left(V_{X1}+V_{Y2}\right)}{V_{X1}+V_{X2}+V_{Y1}+V_{Y2}} \end{array}$$

(2) $$\begin{array}{c} y=\frac{L_{Y}}{2}\frac{\left(V_{X2}+V_{Y2}\right)-\left(V_{X1}+V_{Y1}\right)}{V_{X1}+V_{X2}+V_{Y1}+V_{Y2}} \end{array}$$

where $L_{X}$ and $L_{Y}$ are the dimensions of the PSD sensor and $V_{X1}$, $V_{X2}$, $V_{Y1}$ *y* $V_{Y2}$ are the voltages obtained from the currents of each of the four PSD anodes. The electrical current obtained from the PSD is very low (about nA) and if it was digitized directly, the error would be high. Therefore, each channel (signal of each anode) has been previously amplified by a transimpedance amplifier.

In this work we consider, initially, that the plane of motion of the PSD is parallel to the plane of the emitters and the distance between both planes is calculated from the measurements. According to these considerations, using two emitters, the rotation of the PSD in the horizontal plane can be obtained.

The model of the system used to relate the 3D points of the emitters in the world $\left(X_{e},Y_{e},Z_{e}\right)$ and their impact points on the surface of the sensor $\left(x_{i},y_{i}\right)$ is shown in [26]. Additionally, the authors propose a calibration method to obtain the intrinsic parameters of the measurement system.

The coverage of the system depends on several factors. Firstly, the coverage depends on whether it is necessary to obtain the angle in 3D or 2D. In 2D, as the signal from only two emitters is needed, a higher coverage can be obtained using the same number of emitters as in 3D. On the other hand, it is limited by the detector’s FoV. Depending on the lens used, a higher or lower coverage can be obtained. Moreover, for the same FoV of the receiver, if the ceiling height is increased, the coverage increases. If the height is increased, the signal-to-noise ratio (SNR) decreases. In this case, the signal strength emitted by the emitters must be higher to achieve the same SNR. When implementing this system in large environments, such as airports or shopping malls, it should be taken into account that this system can be used by changing the lamps of existing luminaires for LED beacon bulbs (already developed in our research group of intelligent LED beacon lamps). In addition, in the developments carried out, when there is no beacon–emitter coverage: the position is estimated with inertial sensors and corrected when it enters the coverage of another emitter, but this aspect is not within the scope of this paper.

Different light sources in the environment and not used for positioning (not beacon lamps) do not influence the accuracy of the system. We use amplitude-modulated signals with sinusoidal tone with frequencies of several kHz. The advantage of using these frequencies is that the human eye does not detect fluctuations in illumination (it will not perceive flickering). In addition, conventional LED luminaires are not modulated, they can only have 50, 60, 100 or 120 Hz components depending on the driver used to convert from AC mains to DC. The receiver incorporates filters that eliminate these frequencies. When identifying the signal from each emitter, FDMA is used as indicated in [28], which ensures that there is practically no interference between the emitters and that external light sources do not affect the system. A factor that can influence the precision and accuracy of the system would be the reflections of light in the environment, known as multipath. In previous work, an analysis of the multipath effect in light-based systems were developed. In [27], it is shown that when using AoA, the errors due to the multipath effect can be assumed, something that does not happen with PoA-based systems, for example.

### 2.1. Measurement of the Angle of Rotation Using Two Emitters

It is assumed in this proposal that the emitters are placed on the ceiling (two LED lighting) and that the surface of the PSD and the plane of motion, defined as Z = constant, are coplanar. Considering this scenario, the mobile positioning and rotation with respect to the surface vector can be calculated. If the plane of the emitters is coplanar to the plane of motion, the height between both planes can be obtained from the measurement data.

Let us define the coordinates in the world reference system $\left(X_{e_{1}},Y_{e_{1}},Z_{e_{1}}\right)$ and $\left(X_{e_{2}},Y_{e_{2}},Z_{e_{2}}\right)$ for the two emitters and the coordinates of the point of impact $\left(x_{i_{1}},y_{i_{1}}\right)$ and $\left(x_{i_{2}},y_{i_{2}}\right)$ on the PSD surface for each emitter.

**Figure 3:**
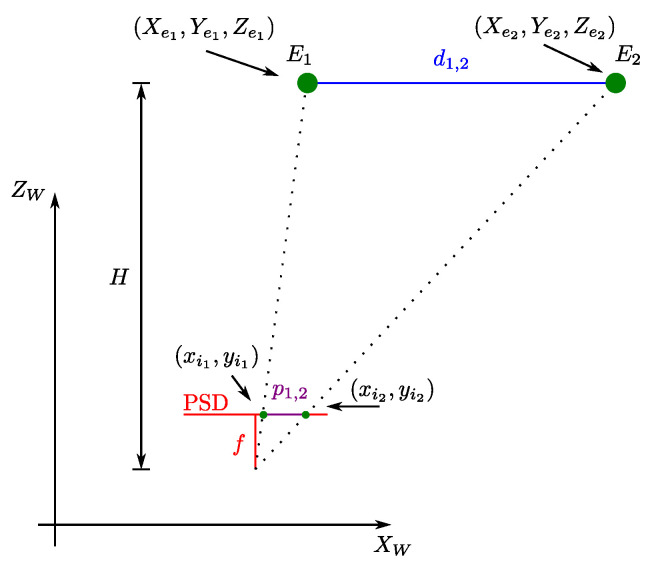
Triangulation method to obtaining the height *H* parameter.

Figure 3 shows the triangulation method to obtain the height *H*. The emitters $E_{1}$ and $E_{2}$ are placed at a distance $d_{1,2}$, giving a projection of $p_{1,2}$ at the PSD surface. The parameter *f* represents the focal length of the sensor (height between the floor and the PSD surface).

**Figure 4:**
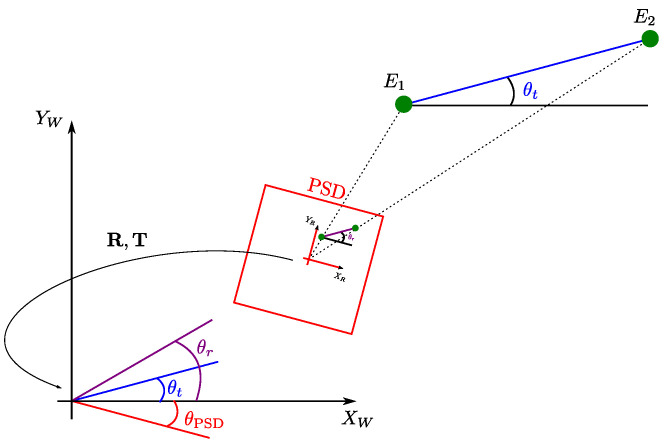
Method for obtaining the angle $\theta_{\mathrm{PSD}}$.

Figure 4 shows the different position parameters. In here, the relation of the angle $\theta_{t}$ between the two emitters along the world reference *x* axis, the angle $\theta_{r}$ for impact points on the PSD surface and the rotation $\theta_{\mathrm{PSD}}$ between the PSD reference system and the world reference system, are presented.

The angle between the two emitters along the world reference *x* axis can be obtained by:

(3) $$\theta_{t}=\mathrm{atan}2\left(\frac{Y_{e_{1}}-Y_{e_{2}}}{X_{e_{1}}-X_{e_{2}}}\right),$$

where atan2 is the arctangent function that gives a modulus 2π output by using the sign of the numerator and denominator to solve quadrant ambiguities. In a similar way, the angle for the impact points on the PSD surface from both emitters along the *x* axis of the PSD reference system can be obtained by:

(4) $$\theta_{r}=\mathrm{atan}2\left(\frac{y_{i_{1}}-y_{i_{2}}}{x_{i_{1}}-x_{i_{2}}}\right).$$

The rotation between the PSD reference system and the world reference system is calculated as:

(5) $$\theta_{\mathrm{PSD}}=\theta_{t}-\theta_{r},$$

In our research, we consider that the motion plane is unknown. To compute the height *H* that separates the emitters plane and the PSD surface plane, the following expression can be used, as depicted in Figure 3.

(6) $$H=f\frac{d_{1,2}}{p_{1,2}}=f\frac{\sqrt{{\left(X_{e_{1}}-X_{e_{2}}\right)}^{2}+{\left(Y_{e_{1}}-Y_{e_{2}}\right)}^{2}}}{\sqrt{{\left(x_{i_{1}}-x_{i_{2}}\right)}^{2}+{\left(y_{i_{1}}-y_{i_{2}}\right)}^{2}}}.$$

### 2.2. Measurement of the Orientation Angles Using Four Emitters

**Figure 5:**
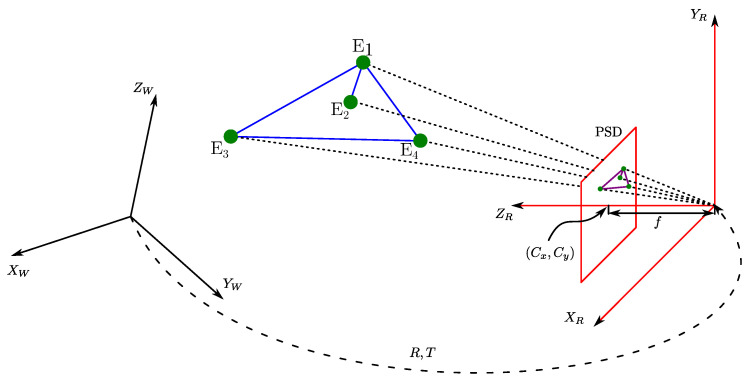
Diagram of the four emitters and their projections on the PSD surface.

To obtain the total orientation, i.e., three angles one for each of the axes, you need to use methods that obtain the total pose of the PSD. The full pose refers to 3D coordinates and three orientation angles. In these cases, it is possible to use positioning strategies that are commonly used in computer vision problems. Such strategies are known as the Perspective-n-Point (PnP) problem. From a set of *n* 3D points in the world and their corresponding 2D projections on the image, or PSD sensor plane in our case (Figure 5), this type of algorithm allows the position and orientation of a calibrated PSD sensor to be determined.

There are several methods to obtain the pose. There are linear methods such as direct linear transformation (DLT) [29] and there are also more complex methods, each with certain restrictions, such as PosIt [30], Coplanar PosIt [31], or CamPoseCalib (CPC) [32].

In this work, we use the DLT method and a joint approach based on DLT and CPC. DLT [29] is a linear method that allows the pose with the information of four emitters to be obtained. With the 3D coordinates of the emitters and the 2D coordinates of their projection points on the PSD surface. A matrix can be created and solved by SVD. From that information, the rotation and translation matrix between the world and PSD sensor coordinate systems is obtained. With the rotation and translation matrix, the coordinates and orientation of the receiver are obtained.

CPC [32] is an iterative method based on the Gauss–Newton algorithm and non-linear least-squares optimization. We have decided to use the CPC method as a trade-off between results obtained, execution time, complexity and use of the least number of points to obtain an accurate solution.

DLT could not provide an accurate solution due to the positioning problem being non-linear. However, it can be used to obtain a roughly approximated solution free of any initialization. Such a solution can in turn be used to initialize the CPC algorithm, increasing its robustness by avoiding possible local minima in the iterative process.

## 3. Experimental Setup

The aim of the experimental tests is to assess the accuracy and precision in calculating the orientation of the agent incorporating the PSD sensor, in different orientations and using 2D positioning methods and 3D positioning methods. When using the 2D positioning method, we only use two emitters and we can only obtain the rotation angle. In this case, different pairs of emitters from which the signal is received at the PSD are used. When using the 3D positioning method, we use four emitters and we can obtain the angles of the three axes.

**Figure 6:**
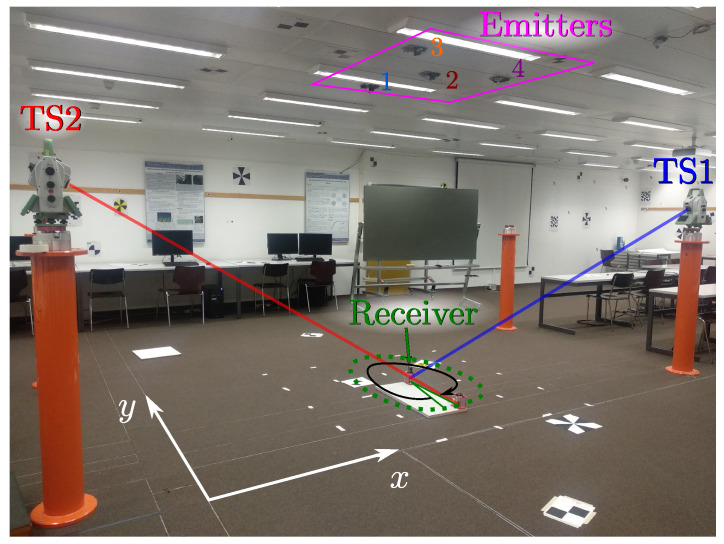
Test environment.

To evaluate the precision and accuracy of the IPS, high-performance total station has been used to compare our results with a more accurate measurement system. Figure 6 shows an image of the test environment. A PSD sensor has been placed below emitter number 2 and rotated at various angles and four spot-shaped light emitters have been placed on the ceiling (magenta square). In Figure 6, the emitters are marked with the numbers 1, 2, 3 and 4. This allows up to six combinations of emitter pairs to be selected. The ground truth was obtained from the combined measurements of two total stations, TS1 and TS2.

The emitter positions to be used are selected as a trade-off between the joint coverage of the pair of emitters and the geometry for the triangulation calculations. With a larger distance among emitters, we will obtain better AoA measurements, but the coverage area will be very narrow as two emitters must be simultaneously seen within the receiver field of view. Each led lamp emits a sinusoidal signal modulated at 6, 8, 10 and 14 kHz.

The receiver used is a Hamamatsu PSD sensor S5991-01 (measured bandwidth: 200 kHz, nominal active area: 9×9 mm${}^{2}$) with the corresponding electronics processing stages for analog amplification. A lens of 7.5 mm of focal length was mounted to form image on the PSD surface.

**Figure 7:**
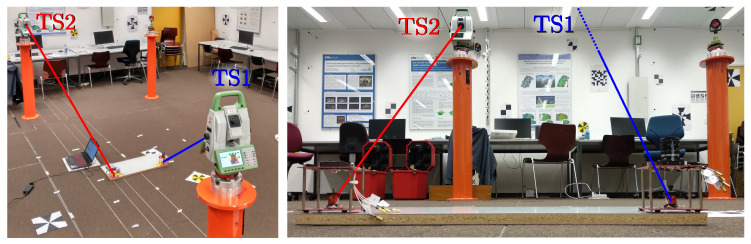
Rotation angle measurements using two Leica TS60 total stations.

The amplified signal of each one of the PSD anodes was acquired with a NI9239 acquisition board (sampling rate: 50 kSamples/s, resolution: 20 bits). The ground truth position and orientation of the receiver was measured with two total stations (Leica TS60) using two 360 mini prisms (Leica GRZ101). Both prisms always maintained their relative position at the base of the PSD on-board plate, which allows us to calculate the rotation (Figure 7). The estimated σ deviations of this equipment are and 0.2 mm in the *x* and *y* axes.

**Figure 8:**
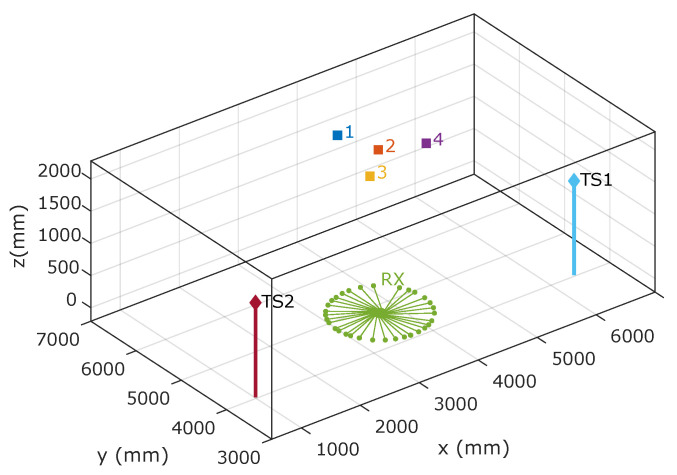
Emitter positions (1–4), receiver (green), and two total stations TS1/TS2 in the environment.

Figure 8 shows the emitter positions and receiver rotation angles within the test environment. It also shows where the total stations that have been used to obtain the ground truth were located.

In order to collect sufficient data to adequately assess accuracy, 15 s time series data were captured for each position. This amount of data allows for adequate reduction of random contributions through averaging for accuracy assessment.

## 4. Results

In this section, we evaluate the precision and accuracy in the determination of orientation angles using the 2D and 3D positioning methods.

### 4.1. Rotation Angle Using Two Emitters

The assessment of the accuracy and precision with two emitters has been performed using the six possible combinations of pairs of emitters. As will be shown below, by selecting the best-placed combinations with respect to the area to be covered, better results can be obtained, but there are no major differences.

#### 4.1.1. Accuracy Assessment

**Figure 9:**
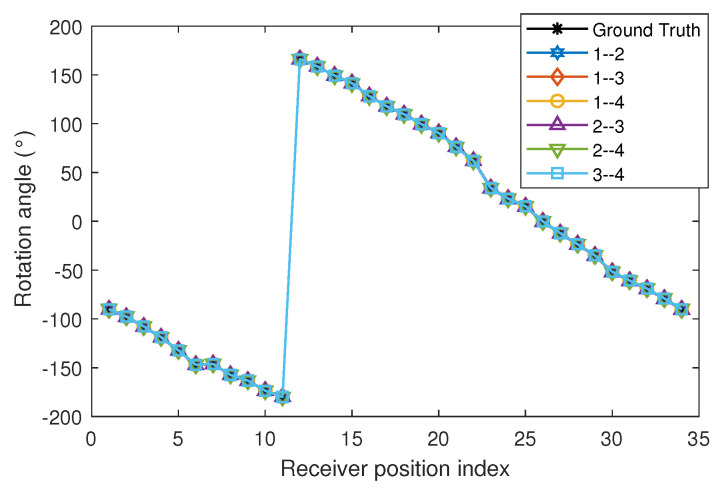
Rotation angle measurements using all combinations of emitter pairs.

The receiver is positioned approximately underneath emitter 2 and is rotated at 34 angles. Measurements were carried out with all possible combinations of emitter pairs. Figure 9 shows the measurements and the ground truth values for all the pair of emitters. The angles of rotation of the measurements were measured continuously, i.e., without large jumps between one angle and the next. Figure 9 shows the value of the angles in the range from −180° to 180°. In the representation there is a jump between the angle close to −180° and the one close to 180°. However, this jump is due to the reference system chosen.

The mean, standard deviation and maximum error values in the determination of the 34 angles of rotation are shown in Table 1. The error is rather small in all cases, mean error smaller than 0.35°·, and we can conclude that the measurement system achieves a very high accuracy in the rotation estimation adequate for most applications. If the last three pairs of emitters with bad geometry with respect to the test surface are excluded, it can be seen that the mean error is close to 0.14°, with a maximum rotation error of less than 0.5°.

**Figure 10:**
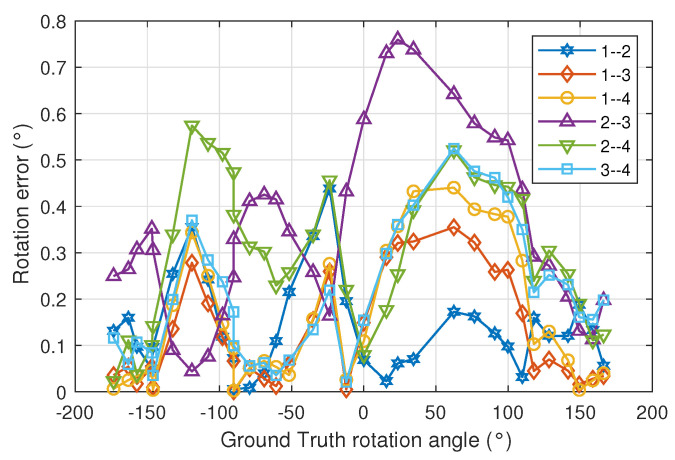
Rotation error as a function of true values of the rotation angles for all combinations of emitter pairs.

Figure 10 shows the rotation error as a function of the true values of the rotation angles for all combinations of emitter pairs. Depending on the geometry of the emitters, the maximum and minimum errors will be obtained at different rotation angles.

**Figure 11:**
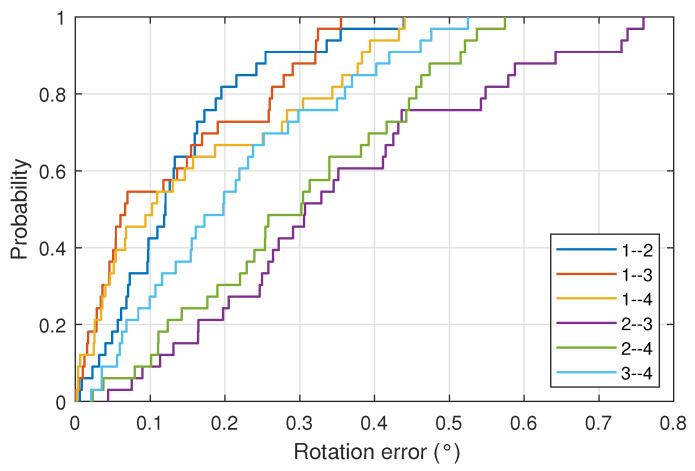
CDF of the rotation error for measurements carried out for all combinations of emitter pairs.

Figure 11 shows the CDF for the measurements carried out with all combinations of emitter pairs. It shows, graphically, the error in the determination of the agent orientation angle for all of the cases.

#### 4.1.2. Precision Assessment

The results have been analyzed in function of the measuring time used to obtain the rotation angle. For each of the angles of the receiver, the variance of the rotation angles were obtained for various measurement time values.

**Figure 12:**
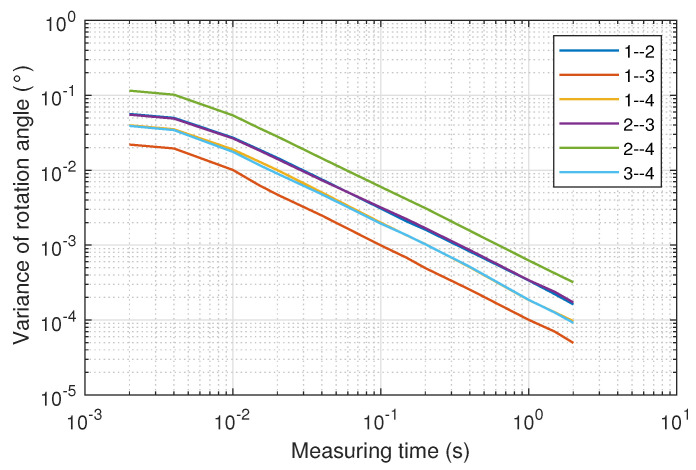
Mean value of the variance of the rotation angles as a function of the measurement time for each combination of the emitters pairs.

Figure 12 shows the mean value of the 34 values of the variance of the rotation angles for each of the combination of the emitters as a function of the measuring time.

In the worst combination of emitters, the variance of the rotation angle is smaller than $6\times 10^{-4}$ degrees when measuring over 1 s, while it worsens to $5\times 10^{-2}$ degrees when the measurement time is decreased to 10 ms. Error measurement results are six times lower when a more convenient combination of emitters is chosen.

### 4.2. Orientation Angles Using Four Emitters

The assessment of the accuracy and precision with four emitters has been performed using DLT and CPC methods.

#### 4.2.1. Accuracy Assessment

As in the previous case, the receiver is placed below emitter 2 and rotates at 34 angles. The difference in this case is that when using 3D positioning methods, the three angles that determine the orientation are obtained, i.e., one for each of the axes. The rotation angle or z-axis angle is the angle used in the previous section. The x- and y-axis angles have a value of 0 degrees since the receiver has been kept parallel to the plane of the emitters in all tests.

**Figure 13:**
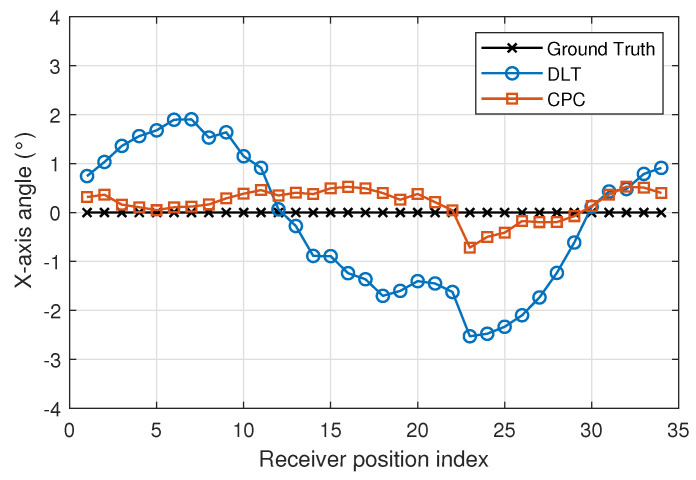
X-axis angle using DLT and CPC.

**Figure 14:**
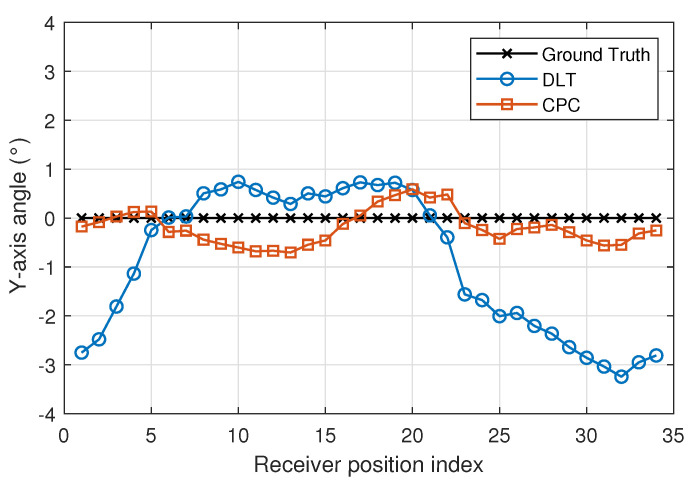
Y-axis angle using DLT and CPC.

**Figure 15:**
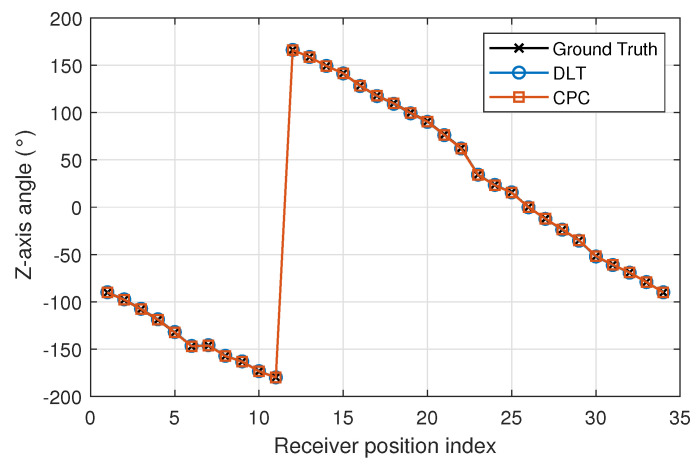
Z-axis angle using DLT and CPC.

Figure 13, Figure 14 and Figure 15 show x-, y- and z-axis angles using DLT and CPC and the true values. The CPC algorithm is initialized by DLT.

Analysing both Figure 13 and Figure 14, the non-zero X and Y angle measurements may be due to the fact that the plane of motion and the plane of the emitters are not coplanar.

**Figure 16:**
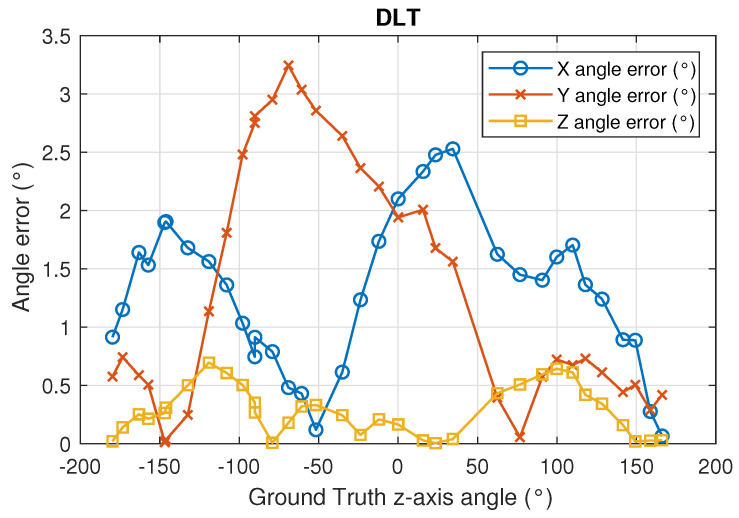
Angle error in the 3 axes using DLT in function of the true value rotation angle (z angle).

**Figure 17:**
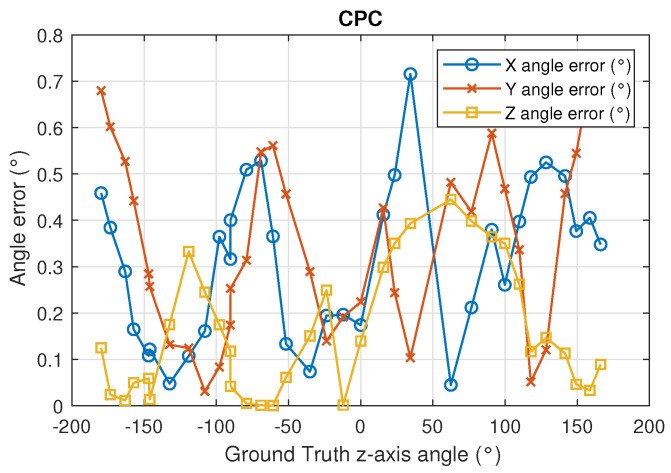
Angle error in the 3 axes using CPC in function of the true value rotation angle (z angle).

The angle error using DLT is higher than using CPC. The Figure 16 and Figure 17 show in more detail how errors vary using DLT and CPC as a function of rotation angle, respectively.

With respect to the DLT method, Figure 16 shows how the angle error in the x- and y-axis is greater than in the z-axis. It can also be seen that the x and y axis angle error is not uniform with the angle of rotation (z axis angle). When the x-axis angle error is minimum the y-axis angle error is higher and vice versa. This may be due to the limitations of the DLT method. Being a linear method and using the minimum number of points necessary, the best solution is not obtained. Even so, the errors are below 0.35 degrees. The error in the z-axis is always less than 0.8 degrees and its behavior does not depend so much on the angle of rotation.

Unlike the DLT method, when CPC method is used, the errors in the measurement of the angles in the three axes are smaller and their behavior is more regular. It should be remembered that the CPC method uses the DLT values as initial values and from them, after several iterations, obtains a better solution. In this case, the errors in the measurement of the angles in the three axes are less than 0.8 degrees.

**Figure 18:**
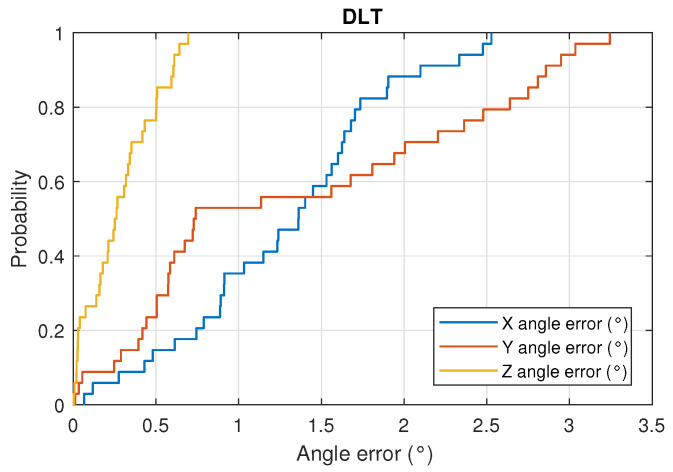
CDF of the angle error in the 3 axes using DLT.

**Figure 19:**
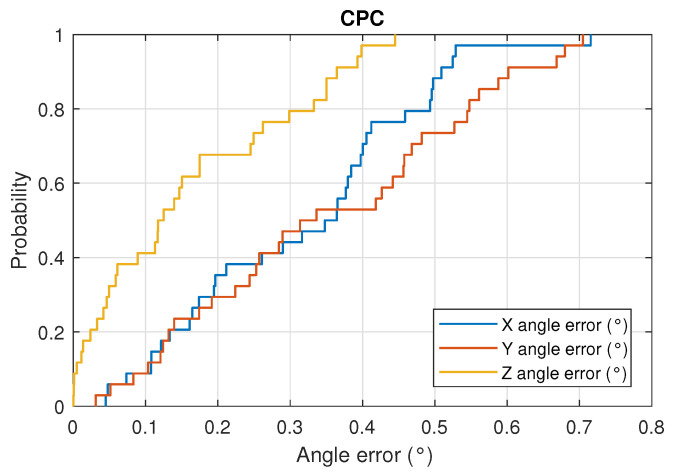
CDF of the angle error in the 3 axes using CPC.

Figure 18 and Figure 19 show the CDF of the angle measurement error in the three axes for the DLT and CPC methods, respectively.

With respect to the DLT method, in 80% of the cases, the z-axis angle error is less than 0.5°, the x-axis angle error is less than 1.7° and the y-axis angle error is less than 2.5°. Using the CPC method, the results are better, obtaining in 80% of the cases that the angle error of the z-axis is less than 0.35°, the x-axis angle error is less than 0.5° and the y-axis error angle is less than 0.55°.

Table 2 shows the mean, standard deviation and maximum values of the angle error in the three axes using DLT and CPC methods.

If we focus on the angle of the z-axis, which in the previous section we called the angle of rotation, the error in determining this angle is similar using the CPC method, which is a 3D positioning method, than using 2D positioning. Remember that if 2D positioning is used, only two emitters are used but it is known a priori that the mobile agent is going to move in a plane parallel to the emitters and only the 3D coordinates and the angle of rotation are obtained (the other two angles are known because the mobile moves in a plane). On the other hand, when 3D positioning methods are used, the four emitters are used but no restriction is imposed and the method has to obtain 3D pose, i.e., three coordinates and three angles.

From the results obtained, with errors in the three axes below 0.55 degrees in 80% of the cases, it is shown that the proposed system is valid for a large number of applications where it is necessary to obtain the orientation of a system or mobile agent with high accuracy.

The computation times of the algorithms used, DLT and CPC, are shown below. The CPC method uses as initial values the results of the DLT method. Therefore, the CPC processing time is the sum of DLT plus the CPC computation time. Using MATLAB and a laptop with an i5-10210U processor we have obtained algorithm computation times of 0.265 ms and 0.615 ms for DLT and CPC, respectively. It should be noted that these times depend on the hardware, programming language used. Moreover, it could be optimized at the final version of the system.

#### 4.2.2. Precision Assessment

As was done for the case of the rotation angle using the 2D positioning method, this section analyzes the precision in the determination of the 3 orientation angles using DLT and CPC. The results have been analyzed in function of the measuring time used to obtain the orientation angles. For each of the 34 angles of the receiver, the variance of the three orientation angles were obtained for various measurement time values.

**Figure 20:**
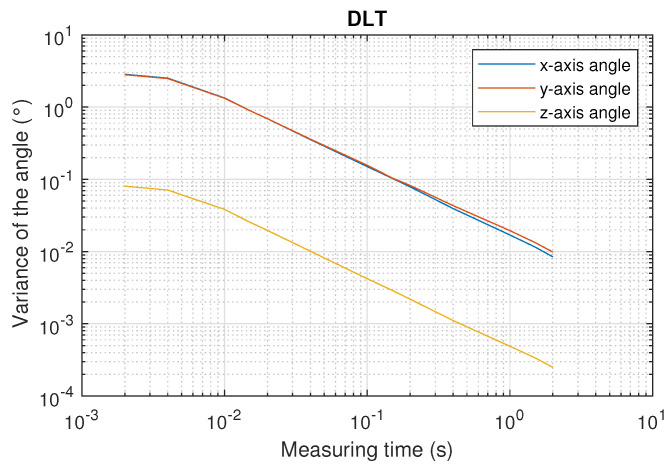
Mean value of the variance of the three axis orientation angles as a function of the measurement time using DLT.

**Figure 21:**
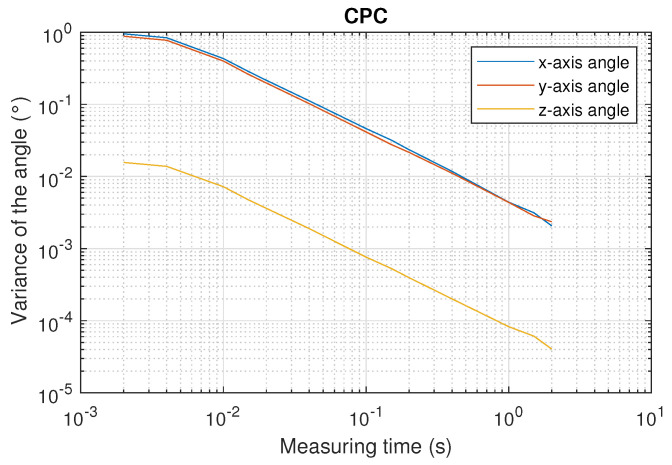
Mean value of the variance of the three axis orientation angles as a function of the measurement time using CPC.

Figure 20 and Figure 21 show the mean value of the 34 values of the variance of the three axis orientation angles using DLT and CPC as a function of the measuring time.

As can be seen from the Figure 20 and Figure 21, using CPC results in variances approximately six times lower than using DLT.

Another important aspect is the difference between the variances in the x- and y-axis angles with respect to the variance in the z-axis. This is because no constraint has been applied when calculating the total pose, i.e., the 3D coordinates and the three orientation angles. Small differences in the x- and y-axis angles can be compensated by small differences in the z-coordinate. However, a change in the z-axis angle rotates the projection of all emitters points on the PSD surface, so that rotation cannot be compensated by variations in the 3D coordinates. This is why better results are obtained in the determination of the z-axis angle.

Using CPC, the variance of the z-axis angle is smaller than $8\times 10^{-5}$ degrees when measuring over 1 s, while it worsens to $7\times 10^{-3}$ degrees when the measurement time is decreased to 10 ms. Error measurement results are six times higher when the x- and y-axis angles are calculated.

A comparison of the results obtained with different the state-of-the-art works has been carried out. The comparison is not direct since each of the works has been carried out under different conditions. We show the results of various light-based IPS systems, in which the error in the determination of the orientation angle is indicated along with the conditions.

In [24], experimental tests were performed in an environment of 2×2 m${}^{2}$ and a height of 3.4 m. They obtained average errors of 5.36, 8.53 and 6.87 degrees in each of the three 3D angles. In the proposal, they used four emitters and a QADA photodiode.

In [23], the authors performed simulations of a 3×5×3 m${}^{3}$ environment for three receiver heights, 0.5, 1 and 1.5 m. They obtained values of errors in the rotation angle (2D) between 6.2 and 8.1 degrees. In this work, two emitters and two photodiodes were needed.

In [21], experimental tests were performed, with a robot moving at 0.2 m/s with a height between emitters and receivers of 3 m. They obtained errors in the rotation angle (2D) of 0.1 rad (5.7 degrees). They used two LED emitters and three photodetectors.

In our proposal, as shown in the results, for an environment of 2.2 m${}^{2}$ and a height of 2.5 m, the average rotation error (2D) is less than 0.2 degrees and the average errors in the three orientation angles (3D) are less than 0.32, 0.35 and 0.16 degrees for the angles in the x, y, and z axes, respectively. In our proposal, two emitters and a PSD photodiode were needed for the case of the 2D rotation angle, and four emitters and a PSD photodiode were required to obtain the three orientation angles. As can be seen, we obtained errors below other state-of-the-art works, using a reduced number of emitters and detectors. The results shown are in an area of coverage somewhat smaller than the works that have been compared. Even so, an increase in the coverage area would not necessarily mean an increase in the error obtained.

Table 3 shows a summary of the comparison with other state-of-the-art works.

## 5. Conclusions

In this work, several experimental tests have been performed in a real environment to assess the precision and accuracy of the proposal to obtain the orientation of the receiver of an IPS based on a PSD sensor. When using the signal received at the detector from two emitters, it is possible to calculate the orientation of the agent (rotation angle) and the separation between the ceiling plane (emitters) and the receiver motion plane with high accuracy, therefore it is possible to correctly position the receiver in the environment. Furthermore, when using the signal received at the detector from four emitters, it is possible to calculate the total pose (3D coordinates and three orientation angles).

Using the signal of two emitters, average errors in the measurement of rotation of approximately 0.15° are obtained using convenient emitter geometries. Precision assessment shows that the variance of rotation angles is smaller than 0.025° when integration time is 2 ms (500 measures of rotation angle per second) with adequate emitter geometries.

Using the signal of four emitters and CPC method mean errors in the measurement of the x-axis and y-axis smaller than 0.35° and of the z-axis angle smaller than 0.16° are obtained. Precision assessment shows that the variance of z-axis orientation angles is smaller than 0.015° when integration time is 2 ms (500 measures of rotation angle per second) and the variance of x- and y-axis orientation angles is approximately six times higher.

Due to the accuracy achieved, this method is very useful in indoor positioning or guidance applications, since knowing the orientation provides added value. It is common to use IMU sensors in mobile devices such as gyroscopes and compasses to know this orientation. The accuracy of such sensors is highly dependent on external factors. For robotics applications in industrial environments, the orientation of the robot at any given time is very important. This type of application is implemented in unmanned aerial vehicle (UAV) systems, industrial tools, automatic guided vehicles (AGVs), forklifts, etc.

## Figures and Tables

**Table 1:** Error values in the determination of the angles of rotation using all possible combinations of emitter pairs.

Pair of Emitters	Error (°)		
	Mean	Standard Deviation	Maximum
1–2	0.133	0.101	0.438
1–3	0.125	0.115	0.355
1–4	0.157	0.145	0.440
2–3	0.342	0.205	0.759
2–4	0.290	0.158	0.574
3–4	0.206	0.144	0.524

**Table 2:** Error values in the determination of the orientation angles using DLT and CPC.

Axis	Error DLT (°)			Error CPC (°)		
	Mean	Std	Maximum	Mean	Std	Maximum
x	1.284	0.638	2.528	0.313	0.167	0.715
y	1.340	1.055	3.243	0.351	0.199	0.704
z	0.279	0.211	0.695	0.158	0.138	0.445

**Table 3:** Comparative with other state-of-the-art works.

Work	Type	Environment	Error 2D Angle (°)	Error 3D Angles (°)	TX and RX
[24]	Experimental	2×2×3.4 m${}^{3}$	6.87	5.36, 8.53, 6.87	4 emitters and 1 QADA photodiode
[23]	Simulation	3×5×3 m${}^{3}$	6.2–8.1		2 emitters and 2 photodiodes
[21]	Experimental	Height 3 m and robot moving at 0.2 m/s	5.7		2 emitters and 3 photodetectors
Ours	Experimental	1.5×1.5×2.5 m${}^{3}$	0.2	0.32, 0.35, 0.16	2 emitters and 1 PSD photodiode

## Data Availability

Not applicable.
